# Influence of the CAD-CAM Systems on the Marginal Accuracy and Mechanical Properties of Dental Restorations

**DOI:** 10.3390/ijerph17124276

**Published:** 2020-06-15

**Authors:** Roberto Padrós, Luís Giner, Mariano Herrero-Climent, Carlos Falcao-Costa, José-Vicente Ríos-Santos, Francisco Javier Gil

**Affiliations:** 1 Barcelona Dental Institute, 08034 Barcelona, Spain; robertopadros@hotmail.com; 2 Faculty of Dentistry, International University of Catalonia, Josep Trueta s/n, 08195 Barcelona, Spain; lginer@uic.es; 3 Porto Dental Institute, 4150-518 Porto, Portugal; dr.herrero@herrerocliment.com (M.H.-C.); cfalcao@ufp.edu.pt (C.F.-C.); 4 Faculty of Health Sciences, Fernando Pessoa University, 4200-150 Porto, Portugal; 5 Department of Periodontology, University of Seville, 41009 Seville, Spain; jvrios@us.es; 6 Bioengineering Institute of Technology, International University of Catalonia, Josep Trueta s/n, 08195 Barcelona, Spain

**Keywords:** CAD-CAM, marginal fit, Cr-Co alloy, dental prosthesis, mechanical properties

## Abstract

The aim of this study was to compare the quality of different computer-assisted-design and computer assisted manufacturing systems (CAD-CAM) generated by only one scanner, focusing on vertical fit discrepancies and the mechanical properties. A master model was obtained from a real clinical situation: the replacement of an absent (pontic) tooth, with the construction of a fixed partial denture on natural abutments with three elements. Nine scans were performed by each tested and 36 copies were designed using a dental CAD-CAM software (Exocad). The frameworks were manufactured using three-axis and five-axis, with the same batch of the chrome-cobalt (CrCo) alloy. The frameworks were not cemented. A focus ion beam-high resolution scanning electron microscope (FIB-HRSEM) allowed us to obtain the vertical gap measurements in five points for each specimen. Roughness parameters were measured using white light interferometry (WLI). The samples were mechanically characterized by means of flexural tests. A servo-hydraulic testing machine was used with a cross-head rate of 1 mm/min. One-way ANOVA statistical analysis was performed to determine whether the vertical discrepancies and mechanical properties were significantly different between each group (significance level *p* < 0.05). The overall mean marginal gap values ranged: from 92.38 ± 19.24 µm to 19.46 ± 10.20 µm, for the samples produced by three-axis and five-axis machines, respectively. Roughness was lower in the five-axis machine than the three-axis one, and as a consequence, the surface quality was better when the five-axis machine was used. These results revealed a statistically significant difference (*p* < 0.005) in the mean marginal gap between the CAD-CAM systems studied. The flexural strength for these restorations range from 6500 to 7000 N, and does not present any statistical differences’ significance between two CAD-CAM systems studied. This contribution suggests that the number of axes improves vertical fit and surface quality due to the lower roughness. These claims show some discrepancies with other studies.

## 1. Introduction

The fabrication of dental prosthesis by using digital processes is getting more popular, thanks to the evolution of the computer-assisted-design and computer assisted manufacturing systems (CAD and CAM) workflows and to a better accuracy of the digital working files. CAD-CAM systems are composed of three main steps: scanning, design and manufacturing [1,2,3,4,5].

The dental prosthesis obtained by casting depends on the experience and ability of the technician. CAD-CAM systems present an important advantage, because they imply a reduced influence of the dental laboratory technician [6,7,8]. It seems that a CAD-CAM workflow favors a better standardization procedure for prosthesis manufacturing. Although these systems require a learning process that may take a significant time as well as an important financial investment and a different management of different materials, it seems that they represent a more viable option in the long-term. Nowadays, there are several CAM techniques that allow the production of metal frameworks, such as metal milling, laser-sintering and wax milling for casting and press techniques. Each one of them represents some advantages and drawbacks. In the dental alloy milling technique, the most distinguished advantage is the ability to work with high quality block alloys. The main problem associated with this CAM technique is its dependence on the numer of axes and the size of the burs used in a milling machine [7,8,9].

In milling or subtraction systems, a machine controlled by a computer is used to mill a block of the desired material for the metal structure. Thanks to software, routines or milling strategies are established that command the milling burs of the machine responsible for the subtraction process, in terms of its direction and magnitude of action [10,11].

For the long-term restoration success, it is very important to take into account several factors, such as the marginal accuracy, fracture stability and material biocompatibility [12,13,14,15,16]. The fabrication systems may influence the characteristics of the finished metal frameworks, such as their surface roughness or their marginal fit [10]. During the manufacturing process, a clinically accepted space or gap can be produced, in the seating of the structure in the abutment, either with natural teeth or implants. Minimizing this space or gap, achieving high precision in the adjustment of the structures, is an important objective in the fabrication process of dental prostheses [17,18,19].

A correct internal and marginal fit will mean less bacterial plaque retention, less gingival inflammation risk, better control of the cementation procedure and lower stress in the framework, overall providing a better clinical result in the short and long term, both for natural teeth and dental implants [20,21]. No consensus was found regarding the size of this gap in different studies; the authors report mismatching data ranging from 35 to 120 μm. These variations result from the technique used for its evaluation or from the study’s design [22,23,24,25,26,27,28].

Consequently, the precision and marginal fit must be carefully observed, since an important marginal fit can contribute to periodontal disease, secondary dental caries, bone loss, or even failure of the dental restoration [29,30,31,32]. It has not yet been determined by the scientific community what is considered an adequate fit or what the size of the clinically accepted space is. Theoretically, the objective would be to achieve a gap between 25 and 50 µm, however discrepancies greater than 120 µm are seen as acceptable in some studies [33,34,35,36,37].

Boitelle et al., in their 2014 systematic review on the fit of prostheses fabricated by CAD-CAM systems, report a misfit in single crowns ranging from 10 to 100 µm, with an internal gap at the level of the axial wall between 23 and 154 µm, and ranging from 45 to 219 µm on its occlusal part. They conclude that it is possible to fabricate frameworks with CAD-CAM systems with a setting less than 80 µm [38].

The different manufacturing systems for metal structures also show differences in their surface finishing; that is, in their roughness, which could have an impact on the retention of these structures [39,40]. An advantage of fabricating structures using CAD-CAM systems is the lesser need for the retouching and adapting of the internal surfaces of the metal framework by the laboratory technician, reducing the manual finishing steps on the inner and outer surfaces of the structure, avoiding its possible deterioration and improving the quality of the final touch of the prosthesis [41,42]. This finishing differs not only between conventional systems for the manufacturing of metal frameworks obtained by casting, but also between different CAD-CAM technologies, the number of axes for milling machines or characteristics and the state of the burs used for milling structures [43]. Some studies show that the number of axes in CAD-CAM has a considerable influence on the machining time for economic reasons, but it is not important in the marginal accuracy and/or surface quality of the dental restorations [1,5,37,40,41,42].

Several methods have been used in order to determine the marginal fits and the adaptation of prosthetic dental restorations: optical microscopy [37], profile projector [43], micro-CT [44] or laser videography [45], with different results. In our contribution, the measurements of these discrepancies are taken by using a high-resolution scanning electron microscope, as it is a much more sensitive method than the traditional ones. Thus, the null hypothesis of this study was that no differences in the marginal accuracy and mechanical properties would be found among the three-axis and five-axis milling system in the manufacturing of a 3-unit metal (Cr-Co) framework and difference in the fit and surface roughness between the two manufacturing systems.

The objective of the present study is to compare the vertical adjustment and surface roughness of metal frameworks made by milling in three-axis or five-axis machines.

## 2. Materials and Methods

### 2.1. Model Preparation

A master model was obtained from a real clinical situation: the replacement of an absent (pontic) tooth (FDI number 15), with the construction of a fixed partial denture on natural abutments (FDI number 14 and 16), with three elements. In order to compare the quality of different CAD/CAM systems, methods were generated by only one scanner (Intra-oral digital scanners (I.O.S.s). Nine scans were performed by each tested I.O.S. and 36 copies were designed using a dental computer-assisted-design/computer-assisted-manufacturing (CAD/CAM) software (Exocad GmbH, Darmstadt, Germany). Eighteen frameworks were manufactured, using 3-axis and 5-axis for each one.

The natural teeth were prepared with a 1.2 mm deep 90° finish line around the contour of the abutments (360°), with a convergence angle to the walls of approximately 10°. The termination line, being natural tooth, was more apical in the vestibular and interproximal areas.

An impression of the abutment teeth was made using an addition silicone 3M Express tm 2 Penta, with double viscosity (3M, Minneapolis, MN, USA). The impression was then poured to obtain a master cast using Diemet-E epoxy resin (Erkodent^TM^ Erich Kopp, Pfalzgrafenweiler, Germany). Subsequently, the master cast was duplicated, obtaining 18 working models.

Duplication procedures were performed in a room with humidity and temperature conditions, within the following limits: 70–80% and 23–25 °C, respectively. All the models were made in Diemet-E epoxy resin by the same operator and following the same working sequence from a silicon index key Wirosil^TM^ Duplicating Silicone (BEGO Herbst GmbH & Co, Bremen, Germany) and following the manufacturer’s instructions. The procedure was repeated until we obtained 18 models free of pores and imperfections.

### 2.2. Study Groups Assignment

The models were divided into two groups:

- Group A: in this group, Cr-Co frameworks were made using a 3-axis milling system. It consists of 9 models.
- Group B: in this group, Cr-Co were made using a 5-axis milling system. 9 models make up this group.

### 2.3. Structure Design and CAD-CAM Manufacturing

The models were digitized with a desktop optical scanner Imetric (Imetric 3D SA (Geneva, Switzerland)), in a dental laboratory (Archimedes Pro^TM^). The structures were designed using a specific software for prosthetics design Exocad^TM^ (Exocad GmbH, Darmstadt, Germany), under the following specifications:

- Thickness of 0.5 mm around the entire outline of the structure.
- Pontic in FPD position number 15, with a convex shape on its cervical surface and 1 mm away from the edentulous crest.
- Application of a space of 50 µm on the dies to 1mm from the location of the finishing line.
- Milling strategies for the machines responsible for milling the structures were established with Hyperdent CAM software (Technology GmbH, Berlin, Germany). Subsequently, the structures were milled with the corresponding milling machines, depending on the group assigned to each model:
  3-axis machine (NX Mach, Siemens, Plano, TX, USA).
  5-axis machine (Zfx-Sauer 10, Zimmer, Dachau, Germany).

The samples were machined with new tools in order to avoid the effect of the possible wear.

**Figure 1:**
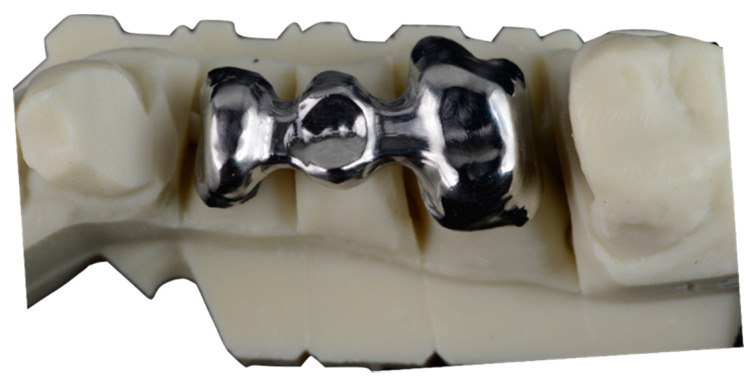
CoCr dental structure studied.

These frameworks were not cemented. The structures were all made of Cr-Co (Figure 1), with the same chemical composition in all cases, which was determined by EDS microanalysis. Cr-Co alloy (Dentaurum Gmbh&Co, Ispringer, Germany) is the most used in the dental restoration using CAD-CAM. One of the most interesting properties of this alloy is its wide melting temperature range, that avoids the distortion of the structures. The chemical composition in weight percentage is shown in Table 1.

### 2.4. Roughness

Roughness measurements were made using white light interferometry (Wyko NT1100 Optical Interferometer, Veeco Instruments, Karlsruhe, Germany), non-contact in vertical scanning interferometry mode, which was used to produce, evaluate and quantify the topography. The interferometric technique is ideal for imaging these surfaces, as a large area of the surface can be imaged with a high vertical resolution (≈2 nm). The analysis area was 124.4 × 94.6 µm. A data analysis was performed with Wyko Vision 32 (Veeco Instruments, Karlsruhe, Germany) which allows us to apply a Gaussian filter to separate waviness and form from roughness. The measurements were made in three different specimens of each type, to characterize the amplitude parameter (S_a_) and the hybrid parameter (index area).

### 2.5. Surface Morphology and Marginal Gap Determination

In this contribution the measurements have been carried out by a Focus Ion Beam-High Resolution Scanning Electron Microscope (Zeiss Neon 4, Oberkochen, Germany), equipped with GEMINIS and J image software for dimensional measurements. The determination of the chemical analysis of the CoCr alloy was done by EDS-microanalysis. More than 5000 measurements of the vertical marginal gap were taken following the procedure described on Holmes et al. [46] for each model. Besides, the load-bearing capacity of the different samples has been evaluated by mechanical tests, in order to evaluate the influence of the marginal fit on the mechanical properties.

**Figure 2:**
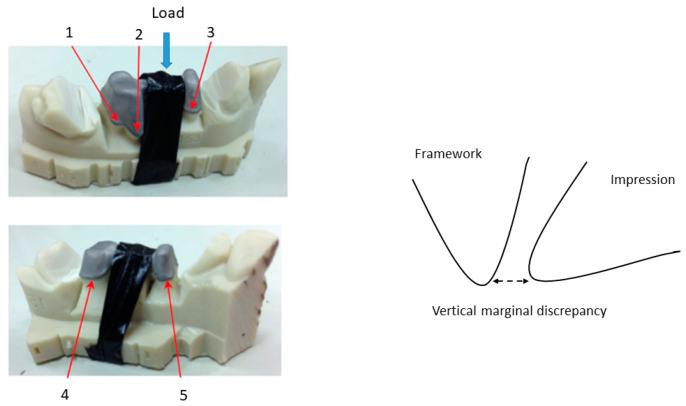
Places of the vertical marginal gap measurements and the point of the load application.

The frameworks were not cemented, and in order to fix the sample in the microcopy chamber, the model is anchoraged with clamps, and in the middle of the model, it submitted under a load of 50 N [46,47]. This load is applied in order to avoid micromovements which could affect the measurements. This load (50 N) does not produce plastic deformation or damages in the model. It can observe that the measurements realized with 20 N and 50 N do not present statistical significance differences between them when the samples do not move. However, the movements of the copper strip of the microscope can produce micromovements when the samples are fixed with 20 N. It is very important to always apply the load in the same position, and to have exactly the same angle between the model and the electron beam. A scheme can be observed in Figure 2. The discrepancies were determined in three places of the buccal part and two places of the palatal. The measurement points are random, but always exactly the same in all samples. Measurements are made at the marked point and the these are taken every 1 µm to the right and to the left, up to a distance of 500 µm from the point to the right and 500 µm to the left. Therefore, for each point, 1000 measurements are taken. These distances and the operation distances are controlled by the software of the microscope. Figure 2 shows the position of each measurement.

**Figure 3:**
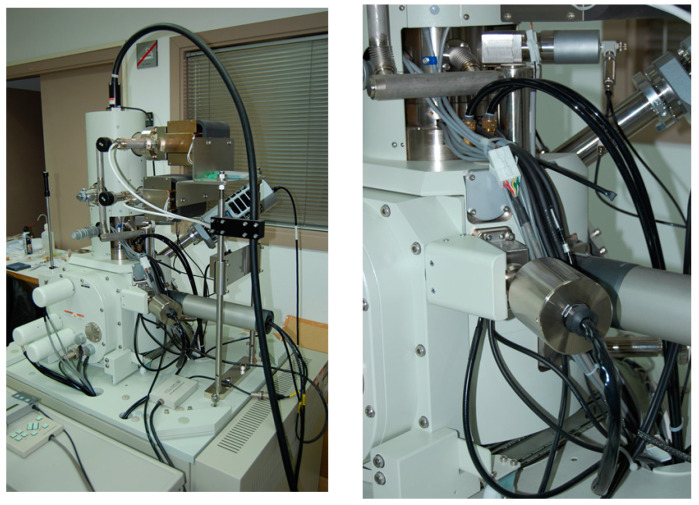
Chamber of the High-resolution scanning electron microscopy and the position and load actuators.

In Figure 3, the chamber of the microscope can be observed with the different actuators, with more detail in the position control and load actuator.

**Figure 4:**
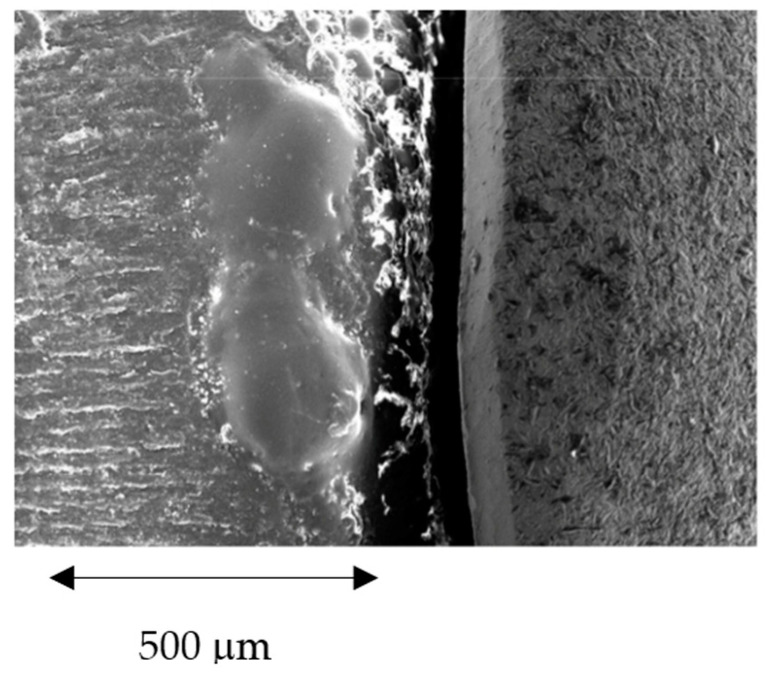
Measurements of the gaps by Scanning Electron Microscopy and image software.

An example of the observation of the gaps; this example is for a model manufactured by 3-axis machine. The model is observed by means of a scanning electron microscope, as can be observed in Figure 4. The software allows for many measurements along the positions established. These values are always checked regarding the position of the model and angle with the electron beam.

### 2.6. Mechanical Tests

**Figure 5:**
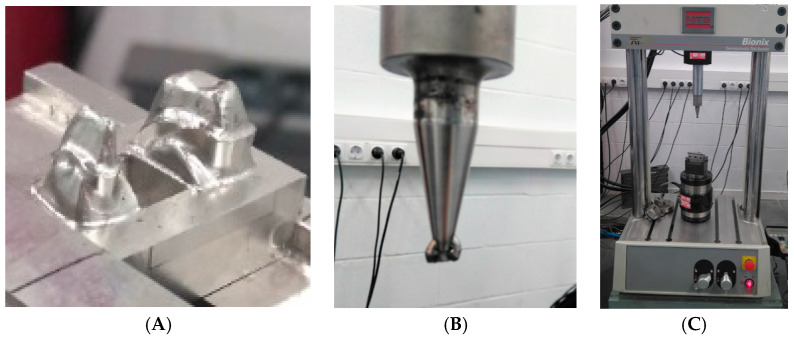
(**A**). Fixation for accommodation of the restoration. (**B**). Tool for applied the load. (**C**). Servo-hydraulic testing machine.

For the flexural strength tests on dental structures, there is no international standard, for this reason, and according to the methods of previous authors we simulate the real case [48,49,50,51]. The conditions can be compared to three-point bending tests. The samples were accommodated in a fixation which was made of refractory steel (material with very high rigidity), in order to avoid the strain of the material of the fixation, as can be observed in Figure 5A. The sample is fixed and it presents a cantilever. In the middle of the restoration process, the load is applied with a pointed tool (Figure 5B). The assays were performed with the servo-hydraulic testing machine MTS Bionix 858 (Figure 5C). This equipment is specially designed for testing biomaterials. This machine was equipped with a load cell MTS of 25 KN. The equipment was controlled by means of a PC equipped with the software TESTAR II^®^. The cross-head rate used was 1 mm/min.

### 2.7. Statistical Analysis

The data was statistically analyzed using t-Student tests and one-way ANOVA tables with Tukey’s multiple comparison tests, in order to evaluate statistically significant differences between the sample groups. The differences were considered to be significant when *p* < 0.005. All statistical analyses were performed with Minitab^TM^ software (Minitab release 13.0).

## 3. Results and Discussion

**Figure 6:**
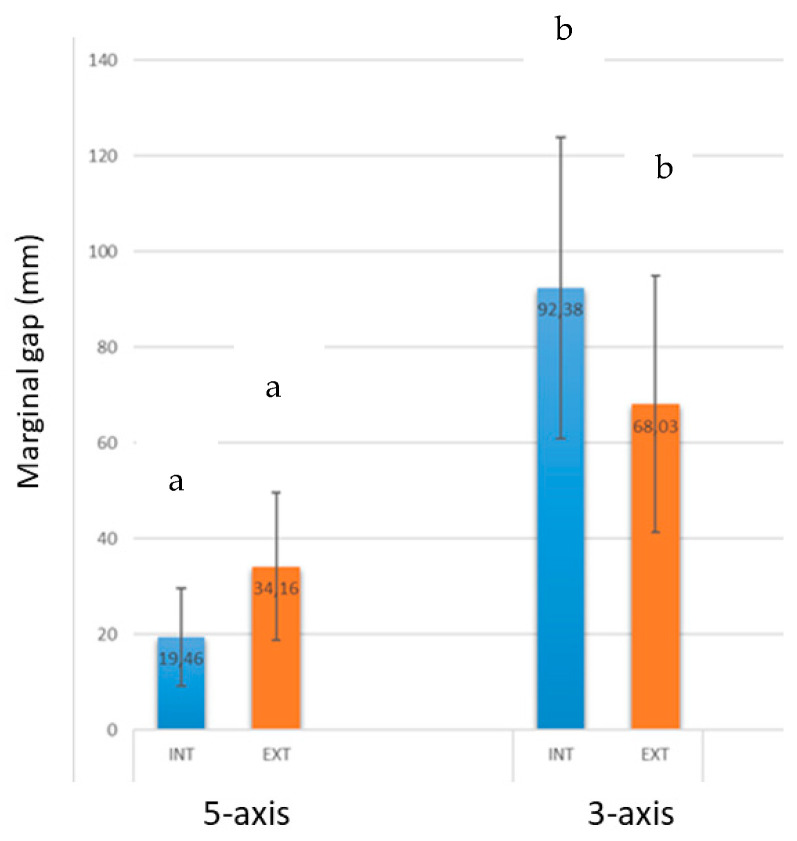
Results of the marginal gaps for the different positions being INT: palatal and EXT Buccal positions for each computer-assisted-design and computer assisted manufacturing systems (CAD-CAM) system. Values with the same letter (a or b) have no statistically significant differences (p<0.005).

The SEM analysis of the marginal gap was performed in 90,000 measurements—i.e., 50,001,125 for each coping, and for each copy, there are five places to measure consequently for each place; there are 1000 measurements—Figure 6 shows the different mean values for the two CAD-CAM systems studied. The results showed a total mean value of 19.46 ± 10.20 µm for buccal positions and 34.16 ± 15.35 µm for palatal positions, corresponding to the restorations made with the five-axis machine. Marginal gaps for the restorations made by the three-axis machine were of 92.38 ± 31.58 µm for buccal positions and 68.03 ± 26.83 µm for palatal ones. The resolution power of the microscope used is 7 nm. The results present statistical significance differences between the two different CAM machines (*p* = 0.0012). However, the differences between buccal and palatal positions for each manufacturer do not present statistical differences.

The five-axis machine presented superior cutting capacities to the three-axis one. This fact improves the milling process and precision, due to the two additional axes, which allow the machining with better reproducibility for the complex parts of this machining to be unachievable when the technicians use a three-axis machine. Consequently, the restorations machined with five-axis equipment present better dimensional accuracy, surface texture and surface finish of the products.

**Figure 7:**
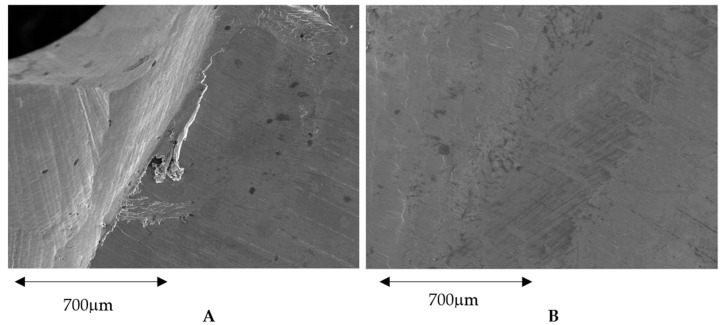
(**A**). Surface obtained by a three-axis machine. (**B**). Surface obtained by a five-axis machine.

In Figure 7A, the surface of the three-axis can be observed, where we can see the machining marks, burrs and other defects in the angled places. In Figure 7B, a surface machined by five-axis is displaying a lower quantity of defects and better surface texture than the three-axis.

**Figure 8:**
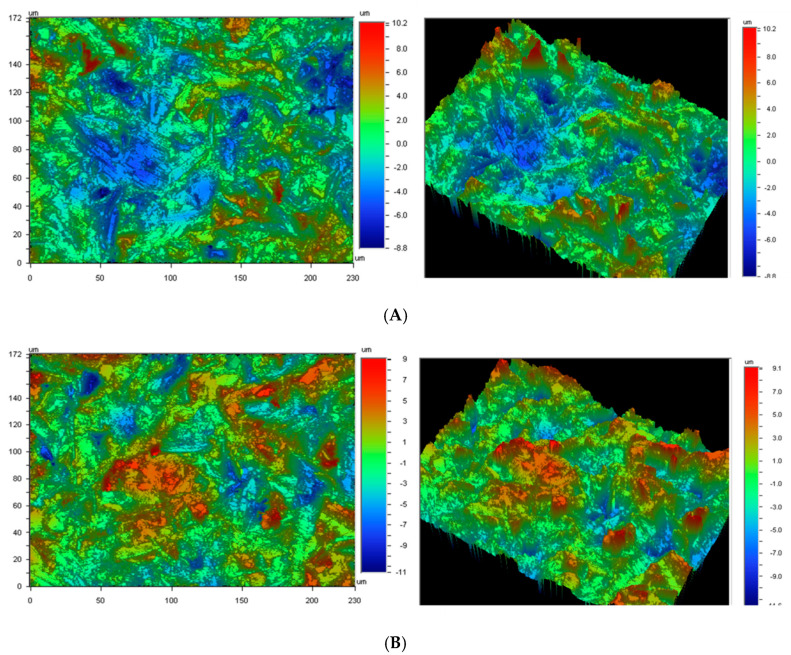
(**A**). Topography of the sample machined with three-axis machine. (**B**). Topography of the sample machined with five-axis machine.

The roughness results confirmed better finished superficial results from the five-axis samples than from the three-axis, as can be seen in Table 2. Figure 8 shows the topography of the three-axis and five-axis restorations.

**Figure 9:**
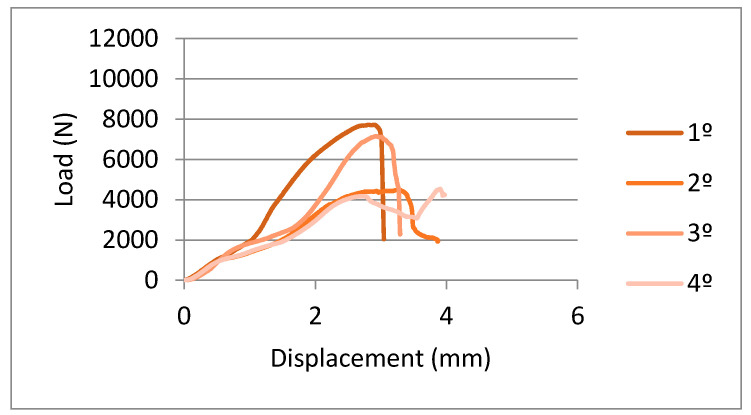
Load-displacement curves for samples obtained by a three-axis machine.

**Figure 10:**
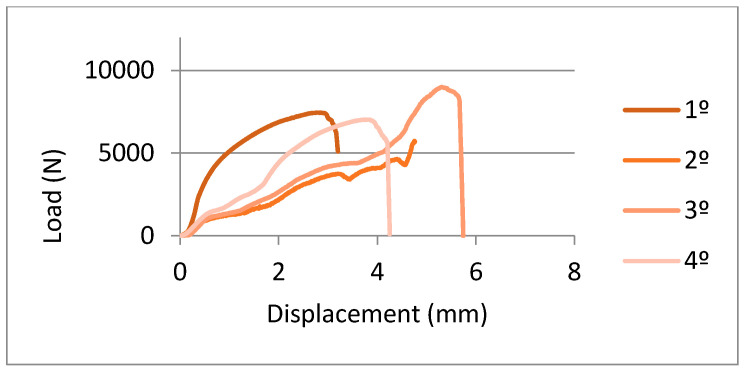
Load-displacement curves for samples obtained by a five-axis machine.

In Figure 9 and Figure 10, you can observe the curves’ force-extension for the flexural tests done for the samples machined with three-axis and five-axis, respectively. From these graphs, we can observe the elastic behavior until 2000–2500 N and higher loads start the plastic deformation of the prosthesis. The mean values of maximum force to fracture were 6578 ± 1500 N and 6978 ± 1289 N. These results do not present statistically significant differences. Consequently, the pieces manufactured by three-axis or five-axis CAD-CAM systems have flexural mechanical behavior that is extremely similar.

No correlation has been found between the vertical gaps obtained by the three-axis and five-axis systems and the mechanical properties. Vertical fits are too small to have an effect on the static mechanical properties. In future studies, the influence of the gap on the cyclic mechanical properties (fatigue) could be analyzed.

**Figure 11:**
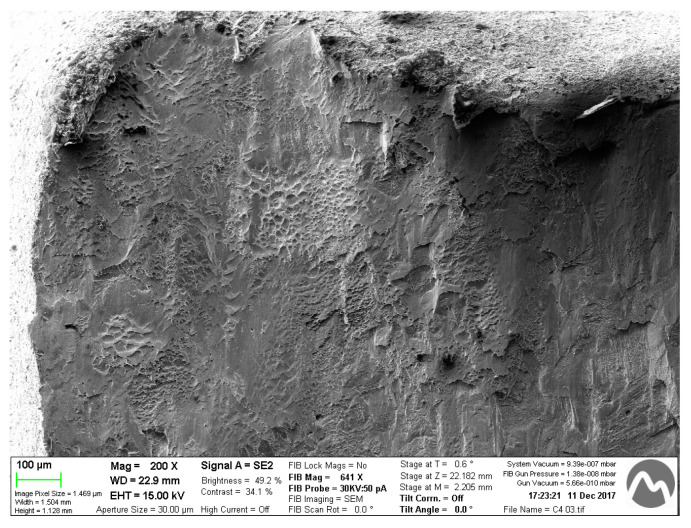
Fractography for a sample obtained by a five-axis machine.

The fractography shows a mixed behavior for two types of samples. There are brittle zones with smooth zones and plastic deformation with dimples and small peaks, as can be observed in Figure 11. In this case, it is a fractography of restoration obtained by a five-axis machine.

In the present study, a non-destructive quantification was performed by field emission scanning microcopy by direct vision, in order to reduce potential distortions generated by cutting and to perform this several times. The material was the same in both cases, with the same chemical composition, same batch and same microstructure. Likewise, the scans were performed with the same equipment and the copies were designed using a dental CAD-CAM software. Consequently, we can confirm that the accuracy and the surface quality of the five-axis are better than the three-axis. Statistical tests revealed a statistically significant difference (*p*-Value < 0.005) in the mean marginal gaps and roughness between copings produced by different manufacturing machines.

In relation to the flexural mechanic behavior, there are not statistically significant differences. The dimensional changes are not sufficient to change the mechanical behavior. A new study could be carried out about the fatigue resistance. It is possible that the better finished surface of the five-axis has a longer fatigue life. The defects on the surface are places where the crack can be initiated more easily [52,53].

The marginal accuracy is one of the most important factors affecting long-term success in fixed restorations. A precise value of an acceptable marginal discrepancy has not yet been defined, however, many authors agree in recognizing a marginal gap less than 120 µm as clinically reasonable [19]. In this study, all mean values of the marginal gap were below 120 µm, regardless of the three- or five-axis machines used.

The structures manufactured with the five-axis milling machines achieve a better fit and a better surface finish, with the benefits that this entails in the behavior of the prosthesis: less bacterial infiltration, better biomechanical behavior, less stress on the abutments, greater retention between others. It is important to know the behavior of the systems that are being used in daily practice in the manufacture of dental prostheses, with their long-term implications.

Karl and collaborators [54] showed that cast frameworks cannot ensure a good passive fit and that CAD-CAM dental restorations have a better marginal fit. Computerized manufacturing technologies have been integrated into dentistry, to improve cost and time efficiency, to provide better accuracy over conventional methods, and to achieve more predictable and sustainable outcomes [6]. CAD-CAM devices in dentistry use subtractive and additive manufacturing technology. In the subtractive method, a cutting device mills out unwanted portions of a block of material to achieve the desired geometry of the object, and a computer program controls all the steps [5]. Thus, the production time of the object decreases, and complex shapes, which are difficult or impossible to manufacture by conventional methods, can be easily created. Unfortunately, this system is quite wasteful, and residual material cannot be reused [55,56].

Few studies comparing the fit of multi-unit FDPs with Co-Cr alloys and different CAD-CAM techniques have been published [7,34,57,58]. These studies are focused on the speed of machining, costs, etc. In this contribution, we have demonstrated that the number of axes is not only interesting for efficiency and economy purposes, it is also very important to the accuracy fit and avoiding the discrepancies and the surface quality. The mean marginal openings of the milled Co-Cr copings studied in this investigation were within the range from 19 for five-axis to 95 for three-axis, considered clinically acceptable by most reports in the dental studies [36]. The internal and marginal fit of restorations is key to the long-term function of FDPs in an oral environment [59]. An ideal marginal fit helps maintain gingival health and hinders the dissolution of the luting cement [20,60]. The long-term clinical success of implant-supported fixed prostheses is critical to the precise fit of any restoration [21]. In the present study, the vertical fit frameworks fabricated by CAD-CAM with five-axis using a CoCr alloy were compared with the three-axis machine and the null hypothesis was rejected.

An advantage of this contribution is the use of a focus ion beam-high resolution scanning electron microscope (FIB-HRSEM), with a resolution power around 7 nm. This measure technique is one of the most sensitive. Besides, the position control and fixing the sample with a load assure the measurement of the vertical gap and making it reproducible. Many measures can be put in place in different places, allowing for a good statistical study. However, this technique is limited by the fact that the internal gap cannot be measured.

The limitations of this study are that it only evaluates two concepts of metal milling machines, although they are two of the most common types in daily practice. Likewise, the structure used is supported only on two abutments, reproducing a structure for a fixed prosthesis on natural tooth with three occlusal units; a structure of a greater length or on a greater number of abutments would represent a more unfavorable situation for its manufacture. On the other hand, the power analysis was done in this study.

## 4. Conclusions

The restorations obtained by a five-axis manufacturer machine present lower marginal gaps than the ones provided by the three-axis manufacturer. The quality surface of the pieces obtained by the five-axis machine have an excellent texture and quality of their surfaces. Roughness results show lower values when using five-axis than three-axis, confirming the excellent finish quality of the samples. The mechanical behavior under flexural loads does not present variations between both. The fractography shows a mixed behavior: brittle and plastic. Further studies are needed to compare the technologies used nowadays in the manufacture of dental prostheses. The rapid incorporation of technology into the dental prosthesis manufacturing process requires verifying the validity of the processes, in view of the wide variety of prosthesis manufacturing options on the market.

## Figures and Tables

**Table 1:** Chemical composition in weight percentage of CoCr alloy used.

Co	Cr	W	Si	C	Nb
56.53 ± 2.11	27.11 ± 1.31	9.64 ± 0.79	1.27 ± 0.80	<1%	<0.1%

**Table 2:** Roughness results obtained for the samples studied.

CAD-CAM	Sa (nm)	SA Index Area
three-axis	809.3 ± 20.8	2.2 ± 0.1
five-axis	779.3 ± 17.8	2.1 ± 0.1
